# The effects of a new shape-memory alloy interspinous process device on the distribution of intervertebral disc pressures in vitro^☆^

**DOI:** 10.1016/S1674-8301(10)60019-X

**Published:** 2010-03

**Authors:** Shengnai Zheng, Qingqiang Yao, Li Cheng, Yan Xu, Peng Yuan, Dongsheng Zhang, Xiangwen Liao, Liming Wang

**Affiliations:** a Department of Orthopaedics, Nanjing First Hospital Affiliated to Nanjing Medical University,Nanjing 210029,Jiangsu Province, China;; b Department of Orthopaedics, Wuxi People's Hospital Affiliated to Nanjing Medical University,Wuxi 214023, Jiangsu Province, China;; c Lab of Biomechanical Laboratory, Shanghai University, Shanghai 200072, China; d Design Center of Shape-memory Alloy Implant, Seemine Ltd, Lanzhou 4616088, Gansu Province , China

**Keywords:** Lumbar spine, disc pressure, interspinous process device, biomechanics

## Abstract

This study was designed to measure the pressure distribution of the intervertebral disc under different degrees of distraction of the interspinous process, because of a suspicion that the degree of distraction of the spinous process may have a close relationship with the disc load share. Six human cadaver lumbar spine L2-L5 segments were loaded in flexion, neutral position, and extension. The L3-L4 disc load was measured at each position using pressure measuring films. Shape-memory interspinous process implants (SMID) with different spacer heights, ranging in size from 10 to 20 mm at 2 mm increments, were used. It was found that a SMID with a spacer height equal to the distance of the interspinous process in the neutral position can share the biomechanical disc load without a significant change of load in the anterior annulus. An interspinous process stabilizing device (IPD) would not be appropriate to use in those cases with serious spinal stenosis because the over-distraction of the interspinous process by the SMID would lead to overloading the anterior annulus which is a recognized cause of disc degeneration.

## INTRODUCTION

The degeneration of the intervertebral disc and facet joint is the main cause of degenerative lumbar spinal stenosis (LSS) with clinical outcomes that include chronic low back pain (LBP) and neurogenic intermittent claudication (NIC)[1]-[5], The pathogenesis of degenerative LSS begins with degeneration of the posterior annulus, advancing to disc herniation and resorption, then to instability with loss of disc height, and finally to stenosis from hypertrophy of the facet joints. Loss of disc height may also cause thickening or “buckling” of the ligamentum flavum at the affected level, contributing to narrowing of the spinal canal[6]-[8].

The conventional treatment for the pain ranges from conservative (nonsteroidal anti-inflammatory drugs [NSAIDs], physical therapy, epidural steroid injection, and bracing) to surgical (decompressive laminectomy with or without fusion and instrumentation)[5],[9]. For patients with severe LSS or ineffective conservative treatment, laminectomy with fusion is the most common surgical method. Although the rate of successful fusion has increased, there has not been a comparable increase in successful clinical outcomes. The main reason is that fusion changes the biomechanical environment-“movement and load”[10]. Recently, several studies have begun to study stabilization of the lumbar spine without fusion, which can stop LBP by improving the load-transfer of the lumbar spine using dynamic stabilization (DS) devices.[11] Compared to other kinds of DS devices, the non-fusion interspinous process stabilization device (IPD) is noteworthy in being minimally invasive surgery (MIS), showing faster recovery and rehabilitation, using local anesthesia during surgery, and having a low complication rate[2],[4],[5],[12].

The studies of the mechanism of IPD are focused on the biomechanical and anatomical changes occurring after the device has been implanted between two adjacent lumbar spinous processes.

Caserta *et al*[13] found that the transverse sectional area of the vertebral canal and lateral neural foramen can be increased at flexion but decreased at extension. Joshua *et al*[14] found that the stenosis of the vertebral canal and lateral neural foramen during extension can be stopped by using an IPD while the vertebral canal and lateral neural foramen of the nearby segments remain unaffected. So anatomically the intent of using IPD is to position the stenotic segment in a slight flexion, and by preventing extension, to relieve the symptoms of LSS, since lumbar flexion may cause improvement of symptoms by increasing the width, height, cross-sectional area of the foramen, and the area at the exiting nerve root, when compared to extension[14]-[15].

In biomechanical tests, the current studies found that the intradisc pressure can be decreased after placement of an IPD. This observation strongly suggests that interspinous process stabilization may be effective because there is good evidence based on both clinical and biomechanical findings that increasing disc pressure may lead to disc degeneration[4],[16].

In the literature, the degree of distraction has always been described as “slight flexion”[5],[17], and there are no studies to investigate the effect of different degrees of distraction of interspinous processes, the lumbar intervertebral disc pressure distribution, and which degree may be the most optimal one. The authors hypothesized that after placement of an IPD, different distraction degrees would cause different changes of disc pressure distribution at the level of instrumentation. The ideal implant may be the one which could significantly decrease the intradisc pressure in the posterior annulus and the nucleus, and redirect a large portion of the load away from the intervertebral disc to the spinous processes in the extension and neutral positions, with no appreciable load change in other parts of the disc at the instrumented level.

**Fig. 1:**
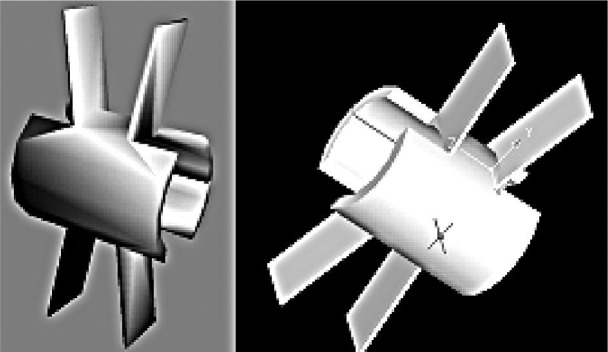
The three dimension CAD shape of the SMID. A: the left-side upper anterior 45-degree view of CAD; B: the right-side upper anterior 45-degree view of CAD.

In this study, a shape-memory interspinous process device (SMID) (Seemine Memory Alloy Inc, Lanzhou, People's Republic of China) was used. This implant is a unitary nickel-titanium alloy IPD comprised of weight-bearing cylindrical spacer and bilateral wings (***Fig. 1***). The cylinder is an elastic weight-bearing structure placed between two adjacent lumbar spinous processes, and the wings which are soft and bend easily at 0°C, resume their primal shape and physical property at 37°C to keep the implant in place. To our knowledge, there are not yet any IPDs made up of nickel-titanium shape-memory alloy in use clinically. In the present in vitro study, the SMID implants were available with 6 spacer heights, ranging from 10 mm to 20 mm at 2 mm increments, allowing us to measure the load distribution in each step during the test.

## MATERIALS AND METHODS

Six cadaver human lumbar L2-L5 spine segments were obtained from fresh human cadavers with a mean age at the time of death of 53 years (range, 45-62 years). The specimens were freshly dissected, sealed in triple plastic bags, frozen, and stored at -28°C until testing. Radiographs were taken before preparation to exclude spinal diseases, damage, and severe degeneration. Specimens without degeneration or with only slight degeneration were used. Each specimen was debrided of muscle and adipose tissue with the ligamentous structures left intact. The average distance between the L3-L4 interspinous processes in the neutral position was 11.2 mm (range, 9.7 mm-11.8 mm), and they were all set to 12 mm by a surgical drill. The cranial (L2) and caudal (L5) vertebral bodies of each specimen were embedded half in polymethylmethacrylate (Vertex Self-curing, Hj Zeist, Netherlands), and the middle disc (L3-L4) was aligned horizontally.

Prior to testing, the specimen was thawed to room temperature (22°C) and then loaded onto a computer-controlled electronic universal testing machine (Zwick-Z010/BIXI, Zwick-Roell, Germany). The machine was capable of applying independent axial loads and bending moments. The specimens were wrapped in a polyethylene sheet to keep them hydrated during the experiment[18].

**Fig. 2:**
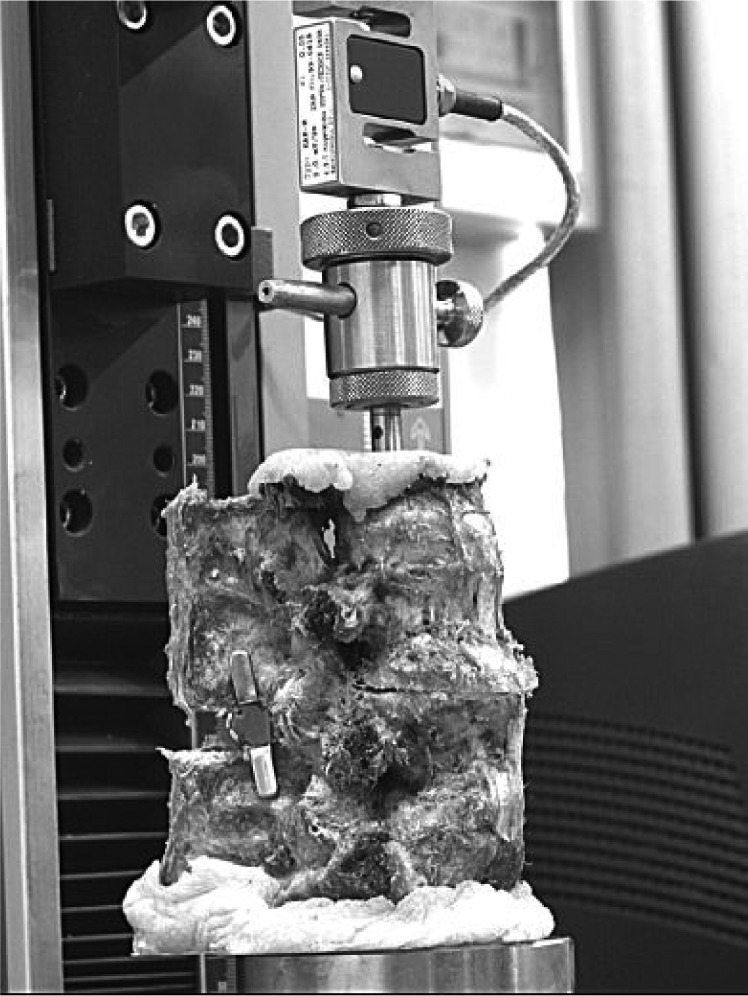
The loading frame and the specimen being test

Before testing, the specimen was placed in a neutral position and a compressive force of 300N was applied for 15 minutes. This technique was performed to precondition the specimens and reduce any postmortem superhydration effects of the intervertebral discs[5],[18]. This was done once for each specimen. Thereafter, a horizontal incision was made in the L3-4 disc using a sharp surgical blade. A pressure measuring film (Prescale Film, Fujifilm, Japan) was implanted into the L3-L4 disc through the incision to measure the load distribution in each step during the test ***(******Fig. 2******)***.

The independent film calibration curve was created in an axial load frame using known applied loads and areas for each film grade used. In this test, the film was of the medium sensitivity type, which ranged from 0.5 MPa to 5.0 MPa. The peak disc pressure was calculated as the greatest pressure (5.0 MPa) from the highest film grade. The average disc pressure was calculated according to the average of the film grade as described by Huang *et al*[19].

**Fig. 3:**
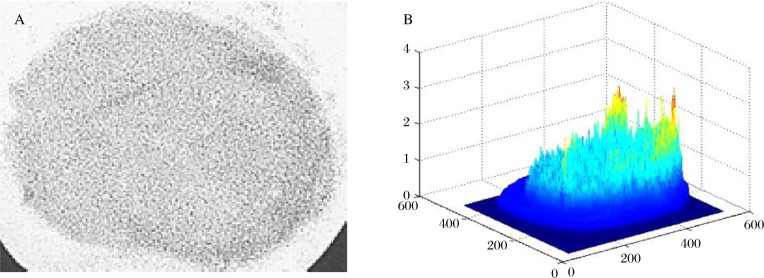
Representative loading patterns of a disc load instrumented with the 12mm SMID in extension. A: the gray scale image; B: the three dimension pressure measuring image in which the film in X-Z plane is in the same position as the film in ***Fig. 3A***. The different heights and colors in the Y plane show different pressures on the disc, with the pressure units of MPa.

After loading, the calibration films were scanned on a flat-bed scanner and converted to 8-bit gray scale images. The images were used to develop gray scale versus pressure calibration data for each film grade using image analysis software (Optical Fringe Pattern Analysis, Shanghai University, China). The data were fit with a third order regression curve that was used to convert the test specimen film loading patterns to pressure and area measurements ***(******Fig. 3******)***.

Each intact specimen was initially placed in the loading frame in the neutral position and subjected to an axial force of 700N for 60 seconds. A 700N force was chosen because it is approximately the amount of force observed in the lumbar spine during sitting and has been used in similar in vitro disc pressure studies. Flexion and extension of the specimen were achieved by applying a bending moment of 7 Nm in the respective direction with a superimposed 700N compressive load[18],[20]-[22].

An SMID implant was placed into icy water for about 10 min to make it soft. Thereafter, the device was placed into the spinous process between L3 and L4. The spacer height of the SMIDs ranged from 10, 12, 14, 16, 18 to 20 mm in size. The 10, 12, and 14mm devices were implanted through an incision in the interspinous ligament after one side of the lateral wings was bent at an angle of about 40 degrees. Six SMIDs of these different sizes were placed into the specimen, respectively. After placement, the specimen was placed in the loading frame again. The temperature of the device was raised to 37°C with a baking light to make the implant resume its primal shape and physical property. The aforementioned sequence was repeated with the specimens loaded in neutral, flexion, and extension positions. A 700 N compressive load was used at every position, and a 7 Nm bending moment was used to create flexion or extension[5],[22]. The intradisc pressure of L3-L4 was measured using pressure measuring film under loading.

## STATISTICAL ANALYSIS

All statistical analyses were performed using the Statistical Package for the Social Sciences (Version 10.0, SPSS Inc., USA). The mean values of average pressures were compared between the intact and implanted specimens using paired *t* tests. Initial analysis involved one way analysis of variance to compare the disc pressure of the intact group and the implanted groups, and repeated-measures analysis using LSD multiple comparisons based on one-way ANOVA to determine which size implant could cause a significant pressure change. All data are expressed as means±SD. Significance of differences was obtained with *P* < 0.05.

## RESULTS

A total of 21 measurements were recorded for each specimen and a total of 126 measurements were recorded in this study.

**Fig. 4:**
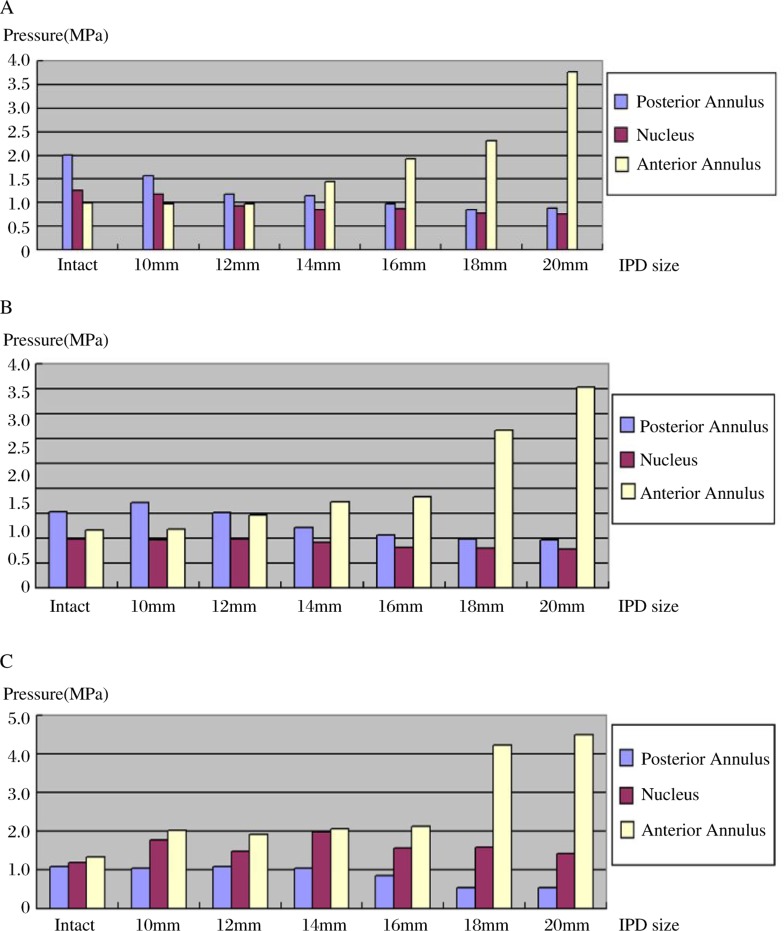
The mean pressures in the posterior annulus, nucleus, and anterior annulus of L3-L4 in extension (A), neutral position (B) and flexion (C). With extension (A) the mean pressure in the posterior annulus was significantly reduced after placement of an implant of ≥ 12 mm in spacer height. There was no significant difference between the mean pressures of the intact nucleus and the implant of less than 14 mm in spacer height. The mean pressure in the anterior annulus was significantly increased after placement of an implant larger than 14 mm. For the neutral position (B): the mean pressures in the posterior annulus were significantly reduced after placement of an implant of ≥ 14 mm in spacer height. There was no significant difference of the mean pressures in the nucleus between the intact and the implant of less than 16 mm, and the mean pressure in the anterior annulus was significantly increased with the spacer height larger than 12 mm. For flexion (C): The mean pressures in the posterior annulus were significantly reduced after placement of an implant of ≥16 mm in spacer height. The mean pressures in the nucleus and the anterior annulus were significantly increased in the intact preparation and in those implanted with a spacer height of ≥ 12 mm.

The distribution of the lumbar intervertebral disc pressure showed a significant change with different degrees of distraction between interspinous processes. ***(******Table 1******,*** ***Fig. 4******)***.

**Table 1:** Mean disc pressure at the L3-L4 level of the intact and SMID implanted specimens

		Posterior Annulus Pressure(MPa)			Nucleus Pressure(MPa)			Anterior Annulus Pressure(MPa)		
		extension	neutral	flexion	extension	neutral	flexion	extension	neutral	flexion
Intact IPD	12mm	2.01±0.61	1.53±0.19	1.08±0.23	1.25±0.54	0.98±0.12	1.18±0.21	0.99±0.21	1.17±0.19	1.34±0.16
	10mm	1.57±0.27	1.72±0.41	1.05±0.21	1.17±0.36	0.97±0.13	1.17±0.19	0.98±0.17	1.19±0.23	1.43±0.21
	12mm	1.18±0.31*	1.51±0.25	1.09±0.14	0.93±0.23	0.98±0.11	1.47±0.11*	0.98±0.11	1.46±0.17*	1.91±0.13*
	14mm	1.15±0.33*	1.21±0.23*	1.05±0.15	0.85±0.21*	0.91±0.14	1.51±0.13*	1.45±0.13*	1.73±0.19*	2.06±0.15*
	16mm	0.90±0.12*	1.06±0.11*	0.85±0.08*	0.86±0.14*	0.81±0.11*	1.57±0.09*	1.93±0.09*	1.84±0.14*	2.12±0.13*
	18mm	0.88±0.07*	0.98±0.13*	0.55±0.07*	0.77±0.09*	0.80±0.09*	1.58±0.11*	2.30±0.08*	3.16±0.13*	4.23±0.09*
	20mm	0.87±0.09*	0.97±0.09*	0.54±0.03*	0.75±0.09*	0.78±0.10*	2.41±0.07*	3.76±0.09*	4.04±0.08*	4.51±0.09*

The SMIDs with the 10 mm spacer height could not share the load on the disc in extension, neutral position, or flexion (*P* > 0.05).

About 46% of the load of the posterior annulus was shared by the implants with a spacer height of 12 mm in extension (2.01±0.61 Mpa, 1.18±0.31 Mpa, *P* < 0.05). The loads of the nucleus and anterior annulus were only slightly increased in flexion, and the distribution of the disc load was not significantly changed.

The segment was slightly flexed with a SMID spacer height of 14 mm. About 47% of the load of the posterior annulus was shared by the implant in extension (2.01±0.61 Mpa, 1.15±0.33 Mpa, *P* < 0.05) and 21% of the load in the neutral position (1.53±0.19 Mpa, 1.21±0.23 Mpa, *P* < 0.05), respectively. The load of the nucleus was also shared in extension (1.25±0.54 Mpa, 0.85±0.21 Mpa, *P* < 0.05) and flexion (1.18±0.21 Mpa, 1.97±0.13 Mpa, *P* < 0.05). The load of the anterior annulus was significantly increased in extension, neutral position, and flexion. The load of posterior annulus was significantly shared by the SMID for the spacer height of 16-20 mm. The load of the anterior annulus was significantly increased in all of the three aforementioned positions, with significantly uneven redistribution of the disc load.

## DISCUSSION

LBP is one of the most prevalent complaints in clinical medicine and is mainly caused by degenerative disc disease (DDD) or LSS. Spinal fusion is the conventional surgical treatment for LBP due to degenerative disorders in the lumbar spine. The new concept of DS is becoming more and more popular since it is thought to relieve LBP by controlling abnormal motions, allowing more physiological load transmission, and prevents degeneration of adjacent segments. Once normal motion and load transmission are achieved, the damaged disc may perhaps repair itself, unless the degeneration is too advanced[4],[23].

There are several types of IPDs for DS, such as the Wallis[24], Coflex[25],[26], X-STOP[5],[17],[27], and SMID. The results of the different types of IPDs are similar in current studies, which show that IPDs may be one of the ideal DS devices for its MIS, disc load sharing, segmental movement restraint, and ability to increase the area of the spinal canal and foramen.[2],[5],[14],[15] Although IPD is one of the ideal DS devices in the treatment of LBP in theory, some pertinent questions about IPD remain to be answered, including how much control of motion is desirable, and how much load should be shared by the implant to unload the damaged disc. Lindsey and colleagues[27] suggested that when the IPD spacer was inserted between the spinous processes of the affected level and the motion segment was placed in slight flexion, the implant could share the disc load and reduce segmental motion in flexion and extension.

Wiseman *et al*.[17] found that an IPD could significantly reduce the mean peak pressure, average pressure, contact area, and force at the implanted level. Swanson *et al*[5] found that an IPD could share the load of the disc without increasing the load on the disc of the neighboring segments. In these studies, the height of IPD spacer was recognized as being of “appropriate size with the implanted segment slightly flexed”. However, the most appropriate size remains controversial. Does the aforementioned “appropriate size with the implanted segment slightly flexed” mean the most optimal size to share load and control segmental motion?

**Fig. 5:**
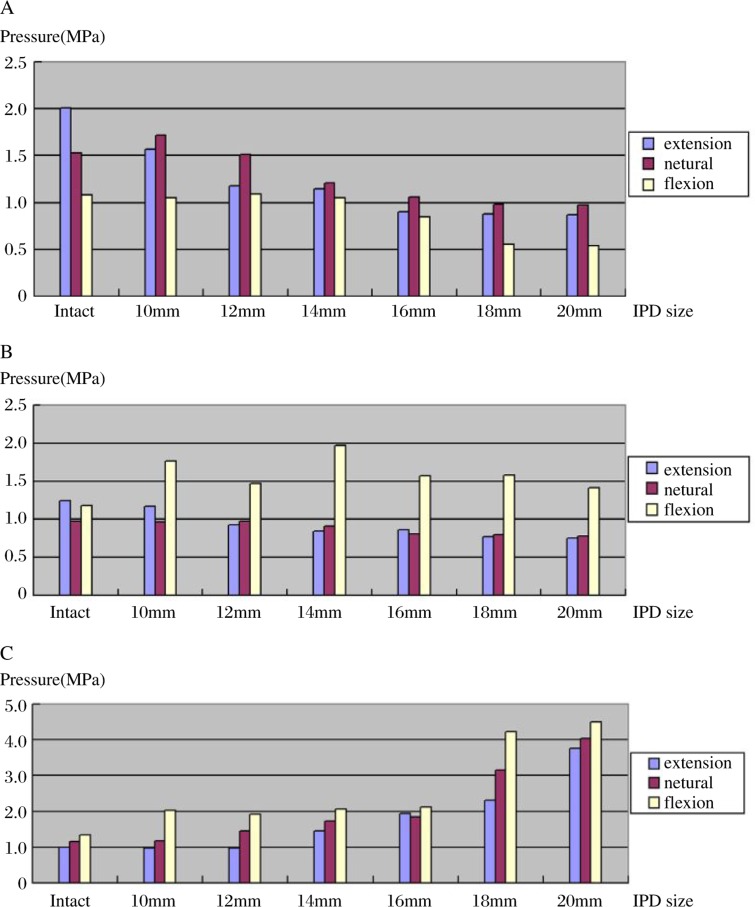
Summary pressures on the posterior annulus (A), anterior annulus (B) and nucleus (C) in motion, and the mean pressure in extension, neutral position and flexion respectively. A: The summary load on the annulus can be significantly shared by the use of an IPD with the spacer height ≥ 12 mm, and there was no significant difference between the intact and the 10 mm implant. B: the summary mean pressures on the annulus in motion and the mean pressure in extension, neutral position, and flexion respectively. The summary load on the anterior annulus can be significantly shared by the use of an IPD. C: the summary mean pressures on the nucleus in motion and the mean pressure in extension, neutral position, and flexion respectively. The summary mean pressures on the nucleus were relative stable when the IPDs with different spacer height, ranging from 10 mm to 20 mm, were implanted.

In our study, the results show that the summary load of the posterior annulus in motion, including extension, neutral position, and flexion, can be significantly shared by the use of the IPD with the spacer height equal to or more than the interspinous processes distance in the neutral position. There is a positive correlation between the spacer height and load sharing ***(******Fig. 5A******)***. In an experiment, Adams *et al*[21] noted a paradoxical decrease in posterior annular pressure during hyperextension at the tested level. They attributed this observation to the facet joints acting as a fulcrum. Thus in our study, it may also be that the IPD with the spacer height equal to or more than the interspinous processes distance in the neutral position can act as a fulcrum in segment motion and redirect the force from the respective posterior annulus to the spinous process. However, in our test the IPD with the spacer height less than the interspinous processes distance in the neutral position cannot share the posterior annulus load in motion, which shows that it fails to be an effective functional fulcrum. Accordingly, the use of an SMID can significantly increase load on the anterior annulus, which shows that there is also a corresponding positive correlation between the fulcrum's height and increasing load ***(******Fig. 5C******)***. But the summary loads on the nucleus in the aforementioned three positions were relative stable when the IPDs with different spacer height were implanted ***(******Fig. 5B******)***. This can be thought of as an effect which McMillan *et al*[28] and McNally and Adams[29] described showing that a normal nucleus is an isotropic structure. Due to fairly constant and slight changes of the nucleus pressure condition, the load distributes uniformly across the endplate.

In our study, the results show that with the use of an IPD with the spacer height less than the interspinous processes distance in the neutral position, the disc load cannot be significantly shared during motion. Clinical and biomechanical studies revealed that increased disc pressure led to disc degeneration, especially in the posterior annulus. Therefore, use of an IPD of this size is not suggested because it does not share the pertinent part of the load.

After placement of the implant with a spacer height equal to the interspinous processes distance in the neutral position, about 46% of the load in the posterior annulus can be shared by the implant in extension. Simultaneously, the load on the nucleus and anterior annulus only slightly increased in flexion after implantation. However, the IPD of this size neither distracts the interspinous process or neural canal. Due to unstretched intervertebral foramina, the symptoms of nerve compression caused by LSS cannot be relieved after surgery. Nevertheless, the IPD of this size is appropriate for those patients with slight LBP caused merely by DDD without the symptom of NIC[30].

After placement of the IPD with a spacer height slightly higher than the interspinous processes distance in the neutral position, the implant is slightly flexed. About 47% of the load of the posterior annulus can be shared by the implant in extension and 21% in the neutral position. The nucleus load is shared in extension and flexion. The anterior annulus load is increased in extension, neutral position, and flexion. Therefore, the distribution of the disc load becomes uneven and the IPD distracts the interspinous process, which leads to the height of the neural canal and intervertebral foramina being slightly increased. Accordingly, this size of IPD is appropriate for those patients with LBP caused by all of the following disorders; disc degeneration, slight stenosis of the neural canal and intervertebral foramina.

After placement of the IPD spacer height obviously higher than the interspinous processes distance in the neutral position, the load of the posterior annulus could be significantly shared in extension, neutral, and flexion positions. The load of the anterior annulus is increased about 400% in these positions. Therefore, the disc load distribution becomes significantly uneven. Although even though the stenosis can be relieved by the higher distraction of the interspinous processes, an implant of this size is not appropriate for the patients with severe degenerative stenosis of the neural canal or intervertebral foramina. A significant load increase on the anterior annulus can accelerate degeneration of the disc.

In conclusion, our study shows that an IPD can act as a fulcrum, and the degree of distraction of the interspinous process caused by the “fulcrum” is correlated with load distribution of the intervertebral disc. In the neutral position, placement of an implant with the spacer height equal to the distance of the interspinous process has a good result in the treatment of DDD. Use of IPD tends to slight flexion of the segment, which is appropriate to relieve LBP caused by slight stenosis of the neural canal and intervertebral foramina. However, the study shows that use of an IPD is not appropriate for those patients with serious LSS because over-distraction of the interspinous process can accelerate disc degeneration by an excessive load on the anterior annulus.
